# Are age-related differences between young and older adults in an affective working memory test sensitive to the music effects?

**DOI:** 10.3389/fnagi.2014.00298

**Published:** 2014-11-11

**Authors:** Erika Borella, Barbara Carretti, Massimo Grassi, Massimo Nucci, Roberta Sciore

**Affiliations:** Department of General Psychology, School of Psychology, University of PadovaPadova, Italy

**Keywords:** music, working memory, emotions, aging, individual differences

## Abstract

There are evidences showing that music can affect cognitive performance by improving our emotional state. The aim of the current study was to analyze whether age-related differences between young and older adults in a Working Memory (WM) Span test in which the stimuli to be recalled have a different valence (i.e., neutral, positive, or negative words), are sensitive to exposure to music. Because some previous studies showed that emotional words can sustain older adults’ performance in WM, we examined whether listening to music could enhance the benefit of emotional material, with respect to neutral words, on WM performance decreasing the age-related difference between younger and older adults. In particular, the effect of two types of music (Mozart vs. Albinoni), which differ in tempo, arousal and mood induction, on age-related differences in an affective version of the Operation WM Span task was analyzed. Results showed no effect of music on the WM test regardless of the emotional content of the music (Mozart vs. Albinoni). However, a valence effect for the words in the WM task was found with a higher number of negative words recalled with respect to positive and neutral ones in both younger and older adults. When individual differences in terms of accuracy in the processing phase of the Operation Span task were considered, only younger low-performing participants were affected by the type music, with the Albinoni condition that lowered their performance with respect to the Mozart condition. Such a result suggests that individual differences in WM performance, at least when young adults are considered, could be affected by the type of music. Altogether, these findings suggest that complex span tasks, such as WM tasks, along with age-related differences are not sensitive to music effects.

Listening to music is not only something we do for our own pleasure; it is also something that affects our behavior. For example, listening to French music induces customers to buy French wines, whereas listening to German music induces customers to buy German wines (North et al., 1997, 1999). The effect of music on behavior is not limited to shopping but also influences our performance in sports (Pates et al., 2003), altruistic behavior (North et al., 2004), and a large variety of human activities (see Schellenberg and Weiss, 2013).

Music also affects cognitive performance. In recent years, one of the most striking and still-debated examples of the effect of music on cognitive performance is the so-called Mozart effect. In the original study, 36 students completed three tests of visuospatial reasoning, one test after each of three different listening conditions (Rauscher et al., 1993). The visuospatial tests were taken from a test of intelligence, each standardized to have the same mean and standard deviation. The experimental condition involved listening to the K448 sonata composed by Mozart. The control conditions involved either 10 min of sitting in silence or listening to relaxation instructions. In order to compare the three listening conditions, the three tests were assumed to be measuring the same thing. In this way, each participant had a score in each of the three conditions. Results showed that performance on the visuospatial tasks was correlated; participants who performed well on one test also performed well on the other two. In particular, performance across tests was significantly higher in the Mozart condition than in the other conditions. This finding became known as the “Mozart effect”.

The Mozart effect had an immediate impact both on the research community and in the broader audience, and the original result has often been misinterpreted even by prestigious newspapers^1^. One of the consequences of this large interest is that the effect has been largely investigated and that, nowadays, the origin of the Mozart effect is largely known. Firstly, the Mozart effect is small, in the sense that the improvement observed in one’s cognitive performance after listening to the music is small (e.g., Nantais and Schellenberg, 1999). Second, the Mozart effect is limited in time: usually, it can be observed within 10 to 15 min after listening. Third, the effect is not Mozart-specific and can be observed with a number of musical stimuli (e.g., Schellenberg and Hallam, 2005; Schellenberg et al., 2007), and even with non-musical stimuli, such as audio books (Nantais and Schellenberg, 1999). Fourth, much of the effect originates from the contrast between the experimental condition (e.g., listening to Mozart) and the control condition (e.g., sitting in silence for 10 min). Nowadays, researchers believe that certain music (such as the sonata K448 by Mozart or other types of music) improves our emotional state (i.e., increases our mood and arousal) in comparison to the control condition and that the improvement observed in the cognitive task is a consequence of the participant’s improved mood (Thompson et al., 2001).

The majority of the results collected in the studies that investigated the Mozart effect concern visual-spatial tasks. There are, however, a certain number of studies that showed that listening to music also improves performance in different types of cognitive tasks, such as memory (Forward Digit Span test) and attentional ones (word fluency) (Mammarella et al., 2007). In the study by Mammarella et al. (2007), 24 older adults started the experiment by listening to a sound stimulus that could be either the “Spring” movement of Vivaldi’s *Four Seasons* or a long-duration white noise track. After 1 min, while the sound stimulus was still playing, participants were asked to complete the forward version of the Digit Span and the Word Fluency tests. Authors also added a control condition under which participants performed both subtests in silence. The results showed that Vivaldi’s music significantly increased memory performance compared with the no-music condition and the white noise condition. The authors suggested that the music increased arousal and made the participants’ mood more positive; therefore, the participants performed better in the music condition than in the remaining conditions.

Noticeably, the performance of older adults in memory tasks can be modulated not only by mood but also by the emotional content of the to-be-recalled material. For instance, although it is well documented that aging coincides with a decline of working memory (WM) (together with other cognitive mechanisms such as inhibition, and processing speed, e.g., Hasher and Zacks, 1988; Craik and Salthouse, 2008; Borella et al., 2011) recent studies found that aging does not seem to hamper the encoding and retrieval of emotional events. In particular, some investigations found that age-related differences between young and older adults in WM performance are attenuated when tasks included valenced words (Mikels et al., 2005; Döhnel et al., 2008). In particular, it has been shown that age-related differences between young and older with positive words were no longer significant, and age-related differences were nullified with negative ones (Mammarella et al., 2013).

In the current study we wanted to extend the findings of Mammarella et al. (2007) using a WM complex span task in which the valence of the stimuli has been manipulated such as in Mammarella et al. (2013): in addition to neutral stimuli (as in a classical WM task), positive and negative ones were also presented (see Mammarella et al., 2013). In particular, we aimed to investigate whether the effect of music on WM performance is limited to music that induces a positive mood or if it can be extended to other types of music. In other words we examined whether listening to a specific mood-type music (e.g., sad or happy) facilitates the encoding (and the subsequent recall) of words that are emotively coherent with the music to which the participant had listened, i.e., the so-called mood-congruency hypothesis (e.g., Mayer et al., 1990). According to the mood-congruency hypothesis, happy material is learned better in happy moods whereas sad material is learned better in sad moods. In the current study, thus, in addition to Mozart’s classic K448 sonata, the participants also listened to Albinoni’s famous *Adagio*. The Adagio is a classic example of sad-sounding music, written with a slow tempo and in a minor key. Further, in the present study participants performed the cognitive tasks *after* listening to the music. There are, in fact, differences between the studies that investigated the effect of music listened *before* a certain cognitive task (e.g., such as the studies on the Mozart effect) and those that investigated the effect of background music; i.e., listening to music *while* involved in a cognitive task. The study by Mammarella et al. (2007) falls between these procedures. Classic theories of cognitive psychology predict that any activity subtracting attentional resources from the cognitive task (e.g., listening to music) should worsen the participant’s performance in the cognitive task (Kahneman, 1973). However, studies that investigate the effect of background music on cognitive performance report mixed results (see Schellenberg and Weiss, 2013, for an overview). Here, to avoid possible confounding in the interpretation of the results, participants performed the cognitive task after they had been listening to the music, such as in the classic Mozart-effect studies. Successively, the effect of the music (Mozart vs. Albinoni) was investigated on an affective version of a classic WM task (i.e., the Operation Span task), wherein the valence of the words was varied (Mammarella et al., 2013) to see whether enhancement due to music is modulated by the valence of the words to be recalled. In particular, this was investigated in groups of younger and older adults to explore the possible role of age differences.

Overall, in line with literature, we expected to find a decline in older adults’ performance in the affective WM task when non-emotional words are considered. In contrast, if music facilitates the encoding and recall of words that are coherent with the mood expressed by the music, as suggested by the mood-congruency hypothesis (e.g., Mayer et al., 1990), we expected to find an interaction between music condition and recall performance in affective words in the two groups that listened to music compared to control groups that listened to a description. In particular, we expected Mozart’s music to enhance the recall of positive words; Albinoni, the recall of negative ones. Alternatively, if the increment in cognitive performance is limited to positive-mood music, we expected to observe the Mozart-effect; i.e., a better WM performance in the Mozart condition (regardless the type of affective words) than in the remaining conditions. Further, music-related effects could also reduce age-related differences between younger and older adults compared to the control groups.

## Method

### Participants

Sixty-three young adults (age range: 20–35 years) and 92 older adults (age range: 64–75) were recruited for this study. The young adults were undergraduate students. The older adults were healthy community-dwelling individuals (for inclusion criteria, see Crook et al., 1986). Older participants were screened for cognitive impairments using the short version of the Italian Checklist for the Multidimensional Assessment (SVAMA) of the elderly used in the Veneto region (Gallina et al., 2006). Only participants who did not report hearing problems and, for older adults, who correctly completed all the items of the SVAMA (Gallina et al., 2006) were included in the study. All participants performed above the cutoff for their age and education in WM-Categorization WM Span test-, and short-term memory- Forward Digit Span, and Backward Digit Span-tasks (see Italian norms; De Beni et al., 2008).

Participants were randomly assigned to three conditions (two music conditions—Mozart or Albinoni—and a control one, in which participants listened to a short story); among these conditions, participants did not differ significantly with regard to years of education and vocabulary (Wechsler vocabulary test, Wechsler, 1981; see Table 1); however, in the negative self-assessment of PANAS (Watson et al., 1988), there was an Age Group effect^2^, with older adults reporting less negative mood than younger adults, *F*_(1,149)_ = 23.71, *p* < 0.001, ηg2 = 0.13 (see Table 1).

**Table 1:** Demographic characteristics of the two age groups (young vs. old) by condition (Mozart vs. Albinoni vs. control conditions).

	Young adults							Older adults						
	Mozart		Albinoni		Control		Mozart		Albinoni		Control	
	(*N* = 23)		(*N* = 20)		(*N* = 20)		(*N* = 31)		(*N* = 31)		(*N* = 31)	
	*M*	SD	*M*	SD	*M*	SD	*M*	SD	*M*	SD	*M*	SD
Age	25.91	4.52	25.20	4.38	25.70	4.91	68.50	3.09	68.67	3.41	68.64	3.51
Education	14.26	2.52	14.50	1.96	14.65	2.16	10.86	2.78	11.33	3.56	11.79	2.99
Vocabulary	45.17	10.39	48.75	8.46	46.50	11.48	46.68	11.71	48.20	12.69	49.79	12.26	
PANAS												
Positive	33.35	6.39	30.20	5.99	33.05	8.49	33.13	5.41	34.32	6.54	33.39	7.00
Negative	23.13	7.03	22.25	6.98	23.80	7.68	17.27	6.44	17.84	6.44	18.52	5.82

### Material

#### Forward digit span and backward digit span tasks (De Beni et al., 2008)

A series of digits were presented at a rate of 1 s per digit; participants had to repeat the digits in the same (forward) or reverse (backward) order. The series started with three digits and rose to nine for the forward task, and went from two to eight for the backward task. Each level contained two strings of digits. After two consecutive recall errors, the task was discontinued. A practice sequence of two digits was given for each task before the test started. One point was awarded for each sequence correctly recalled. The final score corresponded to the total number of correct trials recalled (maximum score 14 for both tasks).

#### Pattern comparison Task (Adapted from Salthouse and Babcock, 1991)

In this task, participants were asked to decide whether arrangements of line segments, presented on two pages, were identical or not. The stimuli for pattern comparison consisted of two pages, each containing one column of 30 items. The stimuli were constructed of three, six, or nine line segments. The items of different difficulty were counterbalanced so that 10 items of three, six, or nine segments were presented on each page. The experimenter used a stopwatch to record the time to complete each page. Three practice trials were given before the experiment started. The dependent variable was the total time taken to complete responses for the two pages.

#### Categorization Working Memory Span (CWMS) Test (Borella et al., 2008)

This task was similar to the classical WM tasks but required the processing of lists of words instead of sentences, which limited the role of semantic processing. The materials consisted of eight sets of words, each set composed of 18 lists of words, organized into series of word lists of different lengths (from 3 to 6). Each list contained five words of high-medium frequency. Lists contained zero, one, or two animal nouns presented in various locations, including the final position. An example of a list is: *house, mother, dog, word, night*.

Participants listened to the lists of words presented at a rate of 1 s per word and were required to tap the table with one hand whenever they heard an animal word (processing phase). The interval between the two lists of words was 2 s. At the end of the series, participants were asked to recall the last word of each list in a serial order (maintenance phase). The experimenter paced the presentation, and two training trials preceded the task. An 85% accuracy criterion on the tapping was required for all participants. The experimenter transcribed tapping errors on a dedicated protocol. The total number of correctly recalled words in the correct serial position was considered the measure of the participant’s WM capacity.

The above tasks, assessing short-term memory, processing speed, and WM, respectively, were used as baseline measures to ensure that participants in the three conditions had a similar cognitive functioning within their age group. Further, for older adults, these measures allowed us to determine that they were aging normally.

#### Affective Operation Working Memory Span Test (Affective Ospan; see Mammarella et al., 2013)

The Affective Ospan required participants to solve a series of math operations (processing phase) while trying to remember a set of unrelated words (maintenance phase). Participants saw one math operation word string at a time, centered on a computer monitor. For each trial, the participant read and solved the math problem aloud and then read the words aloud. Immediately after the participant read the word, the next operation-word string was presented. The operation-word strings were presented in sets of three to six items. Following each complete set, participants were cued to recall target words in the correct order of presentation (e.g., a three-item set: (2 + 5) − 2 = 5, T/F? JOY; (7 − 2) + 3 = 6, T/F? LOVE; (6 − 4) + 7 = 7, T/F? PEACE; Recall: JOY, LOVE, SMILE). Sets of two different lengths (from 3 to 6) were constructed for each affective valence (positive, negative, and neutral). Two trials for each set size were presented, with the order of set size varying randomly, so that participants could not predict the number of items. Target words were selected from an Italian affective word database and were judged in terms of valence and arousal on a 9-point scale. The positive words had a mean valence of 7.8 (1.5) and a mean arousal level of 5.9 (2.8), the negative words had a mean valence of 2.4 (1.8) and a mean arousal level of 5.9 (2.7). Finally the neutral words had a mean valence of 5.5 (1.9) and a mean arousal level of 2.7 (2.3). In order to ensure that participants were not trading off between solving the operations and remembering the words, an 85% accuracy criterion on the math operations was required for all participants. The experimenter transcribed accuracy for math operations on a dedicated protocol. The total number of correctly recalled words in the correct serial position by type of stimulus (neutral, positive, or negative) was considered.

#### Musical excerpts

The musical excerpts consisted of 10 min from Mozart’s (1985, track 1) *Sonata for Two Pianos in D Major*, K. 448, or 10 min from Albinoni’s (1981, track 1) *Adagio in G Minor for Organ and Strings*. The excerpts were transferred from a CD onto the hard disk of a computer without loss of sound quality (44100 Hz sample rate, 16 bits resolution). For the Mozart sonata, we played the entire first movement and replayed it until 10 min were accumulated. The Albinoni adagio was played in the same way; participants heard the entire piece (7 min, 20 s) and a repetition of the early portion.

The control condition consisted of listening to a short description of the invention of television adapted from a standardized test for the assessment of listening comprehension (Carretti et al., 2013), which was selected as an auditory stimulus that would be engaging without being as arousing as Mozart or Albinoni. Both the text and the music excerpts were listened to via Sennheiser HD 280 pro headphones connected to a M-AUDIO Fast Track Pro soundcard.

### Procedure

The experimental procedure described here was in accordance with the Declaration of Helsinki (Sixth revision, 2008). Participants were individually tested in one session (for a total of an hour and a half). At first they filled out a health and demographic questionnaire, the SVAMA, the Wechsler vocabulary subscale, the Forward Digit Span and Backward Digit Span tasks, the processing speed task, and the Categorization Working Memory Span (CWMS) test. After that, depending on the condition, they listened to either the Mozart or the Albinoni (for the music condition groups) or the short text description and then completed the Affective Ospan test and the PANAS. Afterwards, they filled out a brief questionnaire to assess whether they appreciated the music or the short description they had listened to.

To limit the influence of sensory variables (sight and hearing; see Grassi and Borella, 2013) on test results, the auditory presentation was adjusted to the participants’ hearing level. Moreover, for the paper and pencil tasks, all participants were asked whether they found it easy to read the stimuli. All tasks were administrated individually. The order of the tasks within each session was fixed.

## Results

The groups were initially compared in the tasks administered before listening to the music or to the text description with a 2 Age Group (Younger vs. Older adults) × 3 Condition (Albinoni vs. Mozart vs. Control) ANOVA. Results showed that for all measures there was only a main effect of Age Group (Forward Digit Span, *F*_(1,143)_ = 5.94, *p* < 0.05, ηg2 = 0.04; Backward Digit Span, *F*_(1,143)_ = 9.11, *p* < 0.01, ηg2 = 0.06; CWMS recall, *F*_(1,143)_ = 87.03, *p* < 0.001, ηg2 = 0.37; Pattern Comparison times, *F*_(1,143)_ = 55.11, *p* < 0.001, ηg2 = 0.27, Pattern Comparison errors *F*_(1,143)_ = 17.16, *p* < 0.001, ηg2 = 0.11). The descriptive statistics are presented in Table 2. As in the literature, older adults had poor short-term and WM performance and a lower processing speed than younger people. These results confirm the age-related decline in cognitive resources (e.g., Borella et al., 2011).

**Table 2:** Descriptive statistics for the tasks administered before listening to music or text and errors in the secondary task of the Affective Operation span by age groups (young vs. old) and condition (Mozart vs. Albinoni vs. control conditions).

	Young						Old					
	Mozart		Albinoni		Control		Mozart		Albinoni		Control	
	(*N* = 23)		(*N* = 20)		(*N* = 20)		(*N* = 31)		(*N* = 31)		(*N* = 31)	
	*M*	SD	*M*	SD	*M*	SD	*M*	SD	*M*	SD	*M*	SD
Forward digit span	7.22	1.57	7.05	1.70	6.85	1.46	6.13	1.48	6.39	1.38	6.55	1.55
Backward digit span	6.65	2.52	6.10	2.75	5.60	1.54	4.74	1.91	4.90	1.78	5.23	1.91
CWMS-words correctly recalled	33.70	12.64	32.00	8.42	32.00	11.73	18.74	8.26	18.39	8.63	15.42	7.83
Processing speed—times	53.80	9.27	48.23	9.42	50.80	11.42	79.13	24.14	71.98	18.56	75.44	25.91
Processing speed—errors	2.17	2.08	2.55	2.87	2.75	2.61	5.52	4.99	5.29	3.67	4.94	4.13
Affective Operation Span—errors	1.15	0.95	0.74	1.01	0.95	1.15	2.25	1.84	2.00	1.87	1.73	1.82

**Figure 1:**
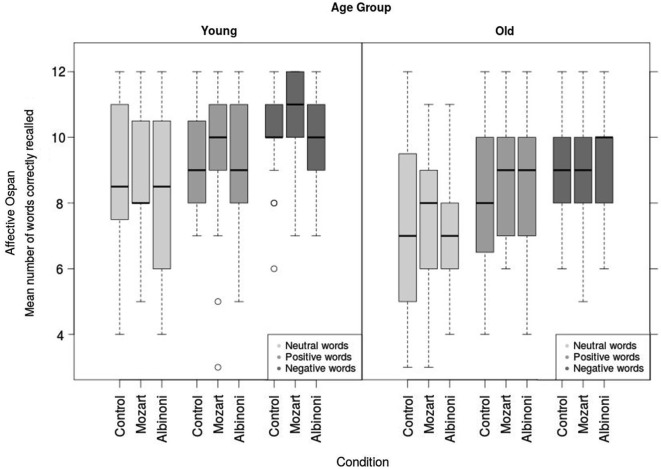
**Affective Operation Span test (mean number of words correctly recalled) as a function of the condition (Mozart, Albinoni music conditions or control one) by age group**.

A three-way mixed design 2 × 3 × 3 ANOVA was conducted with Age Group (Younger vs. Older adults) and Condition (Albinoni vs. Mozart vs. Control) as between-subject effects, and Valence of stimuli (neutral, positive, or negative) as a repeated measure on the Affective Operation Span task. There were significant main effects of Age Group, *F*_(1,143)_ = 19.31, *p* < 0.001, ηg2 = 0.08, with younger adults recalling more correct words than older adults, and Valence, *F*_(1.9,275)_ = 52.65, *p* < 0.001, ηg2 = 0.12. *Post hoc* Tukey tests with Bonferroni Adjusted *p* values revealed that participants recalled fewer correct neutral words than both positive and negative ones (both *p* < 0.001), and participants recalled more negative than positive words (*p* < 0.001). The main effect of Condition was not significant, *F*_(2,146)_ = 0.38, *p* = 0.69, ηg2 = 0.003, i.e., the number of recalled-words was similar across the music conditions. The interactions between factors did not reach significance (Age Group × Valence, *F*_(1.9,275)_ = 0.58, *p* = 0.55, ηg2 = 0.001; Age Group × Condition, *F*_(2,143)_ = 0.41, *p* = 0.66, ηg2 = 0.004; Valence × Condition,* F*_(3.8, 275)_ = 0.32, *p* = 0.86, ηg2 = 0.002; see Figure 1). Overall, the results revealed that younger recalled more words than older adults and that both groups recalled more negative words than positive and neutral words.

Although an 85% accuracy criterion on the math operations on the Affective Operation Span task was required for all participants, the level of performance in the errors in the secondary task was also analyzed. The 2 × 3 ANOVA with Age Group (Younger vs. Older adults) and Condition (Albinoni, Mozart, Control) showed a main effect of Age Group, *F*_(1,143)_ = 18.22, *p* < 0.001, ηg2=0.11, with older adults committing a higher number of errors than younger adults (see Table 2).

Following these results we divided the sample based on the number of errors on the math operation. After computing the median value for the younger and older adult groups separately (0 and 2, respectively), we split the sample of younger and older adults into participants with high and low numbers of errors, and then we ran a two-way mixed design 3 × 3 ANOVA separately for the four groups, with Conditions (Albinoni, Mozart, Control) as between-subject effects and valence of stimuli (neutral, positive, and negative) as a repeated measure on Operation Span task scores.

**Figure 2:**
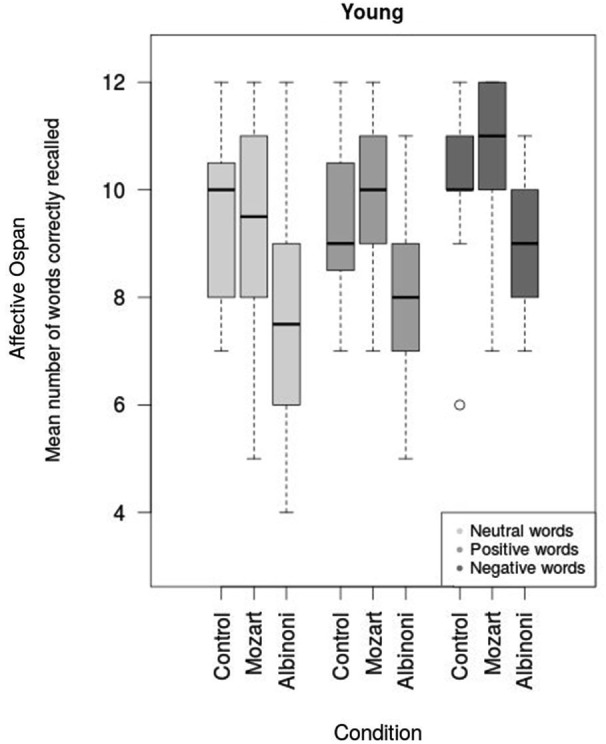
**Performance of low-performing younger adults in the Affective Operation Span test (mean number of words correctly recalled) as a function of the condition (Mozart, Albinoni or control condition)**.

In the younger low-performing group (31 subjects), there were significant main effects of Valence, *F*_(1.9,53.5)_ = 8.21, *p* < 0.01, ηg2 = 0.07 (with the same pattern of results as the previous overall analysis) and of Condition, *F*_(2,28)_ = 3.64, *p* = 0.04, ηg2 = 0.16 (see Figure 2). Tukey tests with Bonferroni Adjusted *p* values revealed that the performance in the Albinoni condition was significantly lower than that obtained in the Mozart condition (*p* < 0.05) but not significantly different from the control condition (*p* = 0.092); the Mozart and control conditions did not differ from each other (*p* = 1). The interactions between factors did not reach a conventional significance level (Valence × Condition, *F*_(3.8,53.5)_ = 0.58 *p =* 0.67, ηg2 = 0.01). Therefore, low-performing younger adults recalled more negative words than positive and neutral words. In addition, the two music conditions gathered different results with more words recalled after listening to Mozart than after listening to Albinoni.

Similar analyses were conducted on the three remaining subsamples, the older low-performing group (27 subjects) and the younger and older high-performing groups (32 and 59 subjects, respectively). They showed exactly the same pattern of results as the first overall analysis; that is, only Valence had a significant main effect with the participants recalling more negative words than positive and neutral words.

It is worth noting that the younger low-performing group did not differ from the younger high-performing group in the negative self-assessment of PANAS—perceived negative mood—*F* < 1 (*M* = 22.45, SD = 6.61; *M* = 23.66, SD = 7.70, respectively), and in the Affective Operation Span task—mean number of words correctly recalled (*F* < 1).

## Discussion

The goal of the current study was to evaluate the effect of listening to two types of music (Mozart vs. Albinoni), which differ in tempo, arousal, and mood induction, on age-related differences between younger and older adults in an Affective WM Span task. As reviewed in the introduction, the impact of listening to music, in particular to the first movement “allegro con spirito” of the Mozart sonata for two pianos in D major (KV 448), is highly debated in the literature. The first data published by Rauscher et al. (1993) demonstrated improved performance on a spatial task (i.e., the Paper Folding test) in college students. Since the publication of this study, further support of the positive effect of listening to Mozart sonata was collected on cognitive performance, along with contrasting results (e.g., Schellenberg and Weiss, 2013).

Starting from this and other results, we explored the possibility that the effect of music, generally attributed to emotional arousal, could be evident in a verbal WM task involving emotional information. It is to note that for the first time, at least to our knowledge, the effect of music in complex span task is examined in the present study. To test this hypothesis, we presented to younger and older adults an affective version of the classical Operation Span (Turner and Engle, 1989), already used by Mammarella et al. (2013), including neutral, positive, and negative words. Using this affective version of the Operation Span task, Mammarella et al. (2013) reported a reduction of age-related differences between young and older adults in WM performance when the recall of emotional words was considered: overall, older adults obtained performance comparable to that of young adults when emotional stimuli (positive and negative) were considered. The age-related difference in WM performance was found for neutral words instead.

In the current study, the Affective Operation Span was presented in three between-subject conditions: The participants carried out the task in the first condition after listening to the Mozart sonata, in the second condition after listening to the Albinoni adagio, and in the third condition after listening to a text on the invention of television. Before listening to music pieces or to the text description, some baseline measures referring to short-term memory, WM, and processing speed were collected.

As expected, the results showed an age-related difference in performance on both the baseline measures and in the affective WM task, favoring younger over older adults. In addition, replicating the results by Mammarella et al. (2013), an effect of valence was found: both younger and older adults recalled a higher number of words with negative valence than positive and neutral ones. As also suggested by Mammarella et al. (2013), negative emotions seem to narrow the focus of attention, leading to overfocusing on target information (e.g., Fredrickson and Branigan, 2005). Since negative emotions foster online processing of detailed information on an item-by-item basis (e.g., Jefferies et al., 2008), this mechanism could be particularly advantageous for serial recall, such as requested by the WM task.

Concerning the effect of experimental manipulation, no effect of the music condition was found; in fact, all three groups obtained comparable performance in the Affective Operation Span task. This was true regardless of the age of the participants and the valence of the words. Therefore, our results contrast with Mammarella et al. (2007) study, as the positive effects of the music did not affect the performance in our WM Span task. However, some differences are worth mentioning between the current study and that by Mammarella et al. (2007): in the latter participants listened to the music during the execution of the memory task, and this could have enhanced the positive effects of music in particular on emotional attitude of the participants. In addition the memory measure used by Mammarella et al. (2007) differed from the present ones in terms of processes required, and this could have affected the results. According to several models of WM, memory span tasks can be distinguished depending on the degree of attentional control required by the task (e.g., Cornoldi and Vecchi, 2003). In this view, complex span tasks, such as that used in the current study, are usually more demanding in terms of attentional resources than simple span tasks, because they require not only to maintain some of the information presented but also to process part of them (in this case solving the mathematic equation). The difference between the types of tasks is evident also considering the development of WM performance across the lifespan. For example, it is now well documented that the decline with aging on complex WM tasks—such as the one we used here—is more accentuated than that found for short-term memory tasks, such as the Digit Span task used by Mammarella et al. (2007) (e.g., Bopp and Verhaeghen, 2005). In this sense, the use of a resource-consuming memory task could have masked or reduced the beneficial effect of music; this could be particularly true in the case of older adults, for whom WM tasks are usually more demanding in terms of cognitive resources compared to short-term memory ones.

However, some interesting results, which require further exploration, regarded the analysis of individual differences. As already mentioned, in the younger subsample, we observed a trend toward a ceiling effect, which could have affected the sensitivity to detect differences due to listening condition. We therefore split the sample according to the accuracy in the secondary task (processing phase) of the Affective Operation Span. The rationale was to examine participants who differed in their efficiency in carrying out the task both in terms of recall and concurrent task and to observe whether lower-performing participants took advantage of listening to music to a higher extent. The analyses highlighted that only in the case of younger low-performing participants did music condition have an effect; in particular, the Albinoni condition lowered their performance with respect to the Mozart condition (the comparison with the control condition was marginally significant). The Mozart condition did not differ from the control, but the performance was very close to the highest performance, therefore we cannot exclude the possibility of underestimating the effect as a result of the ceiling in performance. In the case of the other three groups, the findings replicated the overall pattern of results (i.e., only an effect of valence).

Some hints of a negative effect of the Albinoni condition on cognitive performance have already been presented in a previous study by Thompson et al. (2001): although not statistically significant, in a group of younger adults, performance on a spatial task appeared to be less efficient after listening to Albinoni than Mozart excerpts. In a subsequent study, this result was clearer (Husain et al., 2002): performance on a spatial task was superior after listening to music at a fast rather than a slow tempo and when the music was presented in major rather than minor mode. Therefore, the findings are consistent with the view that the “Mozart effect” is a consequence of changes in arousal and mood. For example, data have suggested that boredom or negative mood can lead to poor performance (O’Hanlon, 1981). However, it is not clear why this was evident only for younger adults and not for the other groups, and further studies are needed to explore this issue.

In general, our findings did not bring strong support to the music effect, at least when emotional stimuli are presented in a complex span task, in line with the literature challenging the robustness of the so-called Mozart effect. For example a recent meta-analysis by Pietschnig et al. (2010) tried to shine light on the consistency of the Mozart effect. The results did not support the existence of a specific performance-enhancing Mozart effect; in fact, the effect size was in the range of a small effect (*d* = 0.37), not dissimilar from the effects of other music conditions not involving listening to a Mozart sonata. In addition, they found that the advantage of the music condition over the nonmusic condition yielded an effect size of 0.15.

One possible reason for the lack of an effect of music on performance could be the fact that the literature seems to suggest that music may have a beneficial effect especially on passive verbal WM tasks (such as Forward Digit Span task) and not on more active tasks (such as the Backward Digit Span task; Steele et al., 1997). As already discussed, the use of a complex memory task (therefore involving not only maintenance processes but also processing requests) could have reduced the potential effect of music on memory performance. Some limitations should be acknowledged; for example, as already mentioned, the WM span task resulted too easy for part of younger adults, with the risk of reducing the sensitivity of the measure used. Secondly, in current study individual differences in terms of participant’s music preference, which has been demonstrated to play a role in explaining the positive effects of music on performance (e.g., Nantais and Schellenberg, 1999), were not considered. For example Nantais and Schellenberg (1999) showed that “Mozart effect” was stronger for those participants who expressed a preference for classical music with respect to those participants who did not. In this sense, our findings could be biased also by individual differences in terms of music preferences within each age group. A third potential limit of the study refers to the lack of a direct measure of mood or arousal collected before listening to music; in the light of the importance of mood and arousal for understanding “Mozart effect”, variations in these parameters in a between-subject design could contribute to reduce the strength of the effect. Finally, future studies should also test whether not only the type of task (complex span task vs. simple span tasks), but also the nature of the task content (verbal vs. visuospatial), could affect the music effect.

In conclusion, the current study did not show any strong evidence of an advantage of music exposure on performance in an affective version of the classical Operation Span task. It is important to note, however, that this is the first study using a complex span task, since most of the study focused on passive memory task (requiring the simple maintenance of information). This warrants some caution in the generalization of the results. A modest effect on performance was found when individual differences were analyzed, with a reversed Mozart effect: younger participants carried out the Affective Operation Span task less efficiently (considering the secondary task) and recalled a lower number of words after listening to sad-sounding music (Albinoni excerpt). No other effects were found in the other groups, so future studies should further analyze the possible role of individual differences.

## Conflict of interest statement

The authors declare that the research was conducted in the absence of any commercial or financial relationships that could be construed as a potential conflict of interest.

## Footnotes

^1^http://www.nytimes.com/1994/08/28/arts/classical-view-listening-to-prozac-er-mozart.html

^2^We conducted several analyses in order to understand whether this difference affected the scores gathered with the Ospan test that are reported later in the manuscript. Negative mood had neither main effects nor interactions with any of the remaining factors.
